# Advanced Practice Clinicians Providing an Increased Share of Primary Care in Skilled Nursing Facilities, 2008-2016

**DOI:** 10.1093/geroni/igaa057.287

**Published:** 2020-12-16

**Authors:** Elizabeth White, Cyrus Kosar, Momotazur Rahman

**Affiliations:** Brown University School of Public Health, Providence, Rhode Island, United States

## Abstract

Rising care complexity in skilled nursing facilities (SNFs), coupled with growing shortages of geriatricians and other primary care physicians able to see SNF patients, have increased demand for nurse practitioners and physician assistants (NP/PAs). We used 2008-2016 Medicare Part A and B claims and nursing home assessment data to describe longitudinal trends in NP/PA practice in SNFs. We identified 8,877,094 SNF post-acute primary care visits for 1,494,113 Medicare beneficiaries. The total number of visits increased from 850,285 in 2008 to 1,189,553 in 2016. The share of visits by NP/PAs rose significantly over time, from 24% of visits in 2008 to 43% in 2016. 71% of SNFs (n=10,139) used NP/PAs in 2016, up from 46% (n=6,696) in 2008. The number of NP/PAs practicing in SNFs more than doubled, from 4,472 clinicians in 2008 to 10,000 in 2016. The number of physicians practicing in SNFs declined from 26,297 in 2008 to 19,745 in 2016. NP/PAs represented 14% of all SNF medical providers in 2008 and 34% of providers in 2016. In 2016, 48% of NP/PAs were SNFists (i.e. >90% of visits billed in SNF), vs. only 11% of physicians. SNFs with NP/PAs are on average larger, more likely urban, for profit, and care for larger populations of racial minorities, than SNFs without NP/PAs. SNFs with NP/PAs also have more short-stay Medicare residents, more admissions, higher nurse and rehab staffing levels, and higher case mix. These findings show that NP/PAs are taking on increasingly prominent roles as medical providers in SNFs.

